# New Inventions

**Published:** 1912-11

**Authors:** 


### NEW INVENTIONS

---

Reported especially for this paper by H. B. Willson & Co., Patent Attorneys, 715 Eighth Street N. W., Washington, D. C.

---

A Complete Copy of any of these patents will be forwarded to any person by Messrs. Wilson & Co., on receipt of ten cents. Persons ordering Copies must give Number of Patent.

---

J. M. Francis, Detroit, Mich., assignor to Parke, Davis & Co., Detroit, Michigan, 1,039,114, Package.

---

R. P. Barnstead, Boston, Mass., 1,039,243, Sterilizer.

---

Otto Schonherr, Christiana, and Johannes Brode, Christiana, Norway, assignors to Norsk-Hydro-Elektrisk Kvaelstofaktieselskab, Christiania, Norway, 1,039,325, Process of Making Nitrates and Nitric Acid.

---

W. D. C. Prideaux, Weymouth, Eng., 1,039,591, Hypodermic or other Syringe.

---

F. A. Denquer, New York, N. Y., 1,039,708, Physicians' Table.

---

Wm. Diemer, Toledo, O., assignor to Gendron Wheel Co., Toledo, O., 1,039,709, Invalid's Chair.

Anton Weindel, Leverkusen, near Cologne, Ger., assignor to Farbenfabriken Vorm, Friedr. Bayer & Co., Elberfeld, Ger., 1,-039,858, Process for Producing products from Phonols with Formic Aldehyde.

Albert Wolff, Cologne, Ger., assignor to (Mrs.) Marid Steinkruger, nee Engelskirchen, Cologne, Bickendorf, Ger., 1,-039,875, Preparation of Formic Esters.

J. W. Allan, Omaha, Neb., 1,039,877, Combination Syringe Case and Supporter.

Alfred Klotch, Munich, Ger., 1,039,962, Hot Air Bath.

A. J. Petter, Los Angeles, Cal., 1,040,238, Bathing Apparatus.

Gustave Fernekes, West Elizabeth, Pa. Assignor to J. A. Snee, West Elizabeth, Pa. 1,038,547. Method of Producing Formalde-hyde.

J. V. McManis & W. L. Sherwood, Kirksville, Mo. Said Sherwood, assignor to said McManis. 1,038,619. Osteopathic Table.

W. T. Merchant, Louisville, Ky. 1,038,623. Hospital bed.

G. M. Rhone, Brownwood, Tex. 1,038,960. Syringe.

